# TcI/TcII co-infection can enhance *Trypanosoma cruzi* growth in *Rhodnius prolixus*

**DOI:** 10.1186/1756-3305-7-94

**Published:** 2014-03-04

**Authors:** Catarina A C Araújo, Peter J Waniek, Ana M Jansen

**Affiliations:** 1 Laboratório de Biologia de Tripanosomatídeos, Instituto Oswaldo Cruz – IOC/FIOCRUZ, Av. Brasil 4365, 21045-900 Rio de Janeiro, Brazil

**Keywords:** *Trypanosoma cruzi*, TcI, TcII, Mixed infections, *Rhodnius prolixus*, Digestive tract

## Abstract

**Background:**

*Rhodnius prolixus* is an obligate haematophagous insect and one of the most important vectors of *Trypanosoma cruzi*, the causative agent of Chagas disease in the Americas. *T. cruzi* is a highly variable parasite which is not transmitted in the same efficiency by the different triatomine vectors. Because different *T. cruzi* genotypes are aetiopathologically divergent, further elucidation of the transmission abilities of different Chagas disease vectors is extremely important.

**Findings:**

In the present study, the growth behaviour of two *T. cruzi* isolates, MDID/BR/1993/C45 (TcI) and TBRA/BR/1999/JCA3 (TcII), sharing the same microhabitat (intestinal tract) in single and mixed infections, was examined. The distribution patterns and parasite population densities were evaluated at 7, 14 and 21 days after feeding (daf) by quantification of parasites using Neubauer haemocytometric measurements and mini-exon PCR to identify TcI and TcII subpopulations. Parasitic colonization in the small intestine was more successful in the mixed infection model than the single infection models at 21 daf. In the rectal lumen and wall, the growth behaviour of the mixed infection was similar to that of the TcI group, although the total parasite number was lower. In the TcII group, no metacyclic trypomastigote forms were found. PCR analysis of the contents of each dissected region showed different genotype fractions in the mixed infection model, in which TcI seemed to be the predominant isolate.

**Conclusion:**

The different growth behaviour of the TcI and TcII isolates in single and mixed infection models demonstrated that possibly an intraspecific factor modulates parasitic development in the intestine of *R. prolixus*.

## Findings

### Background

In the Amazon region *Rhodnius prolixus* (Hemiptera, Reduviidae) is the main vector of *Trypanosoma cruzi* (Kinetoplastida, Trypanosomatidae), the aetiological agent of Chagas disease, which circulates between sylvatic mammals and triatomines [1,2]. *R. prolixus* is one of the most widespread triatomines, frequently found among other vector species in Central and northwest South America and is considered the main vector of *T. cruzi* in Venezuela, Colombia and parts of Central America [3]. This triatomine species is commonly found in association with palm trees and their related fauna [3-5].

The genetic polymorphism of *T. cruzi* initially led to a division into three divergent lineages, known as *T. cruzi* I (TcI), *T. cruzi* II (TcII) and zymodeme 3 – a group that fit neither in TcI nor TcII [6]. Although additional subdivisions of the *T. cruzi* group (IIa–e) have been proposed [7,8], a new classification (TcI–TcVI) has been recently adopted [9].

Mixed parasitic infections in a single host are common in nature in both mammalian and insect hosts, and can be considered a result of multiple sequential infections by feeding of the insect vector on different vertebrate hosts and/or parasite intake via coprophagy, which is a normal behaviour of triatomines [10-12]. Moreover, *R. prolixus* is regularly found in nature concomitantly infected with *T. cruzi* and *Trypanosoma rangeli*[13]. To date, *Rhodnius* is the only genus from which infective forms of *T. rangeli* in the salivary glands have been reported [14].

Intra-host competition and cooperation between parasites from different genotypes accelerates natural selection and has been described as an important factor that influences their ecology, virulence, and evolution [15,16]. Parasitic co-infection of a single host may also lead to a competition for resources and the parasite-host system affects parasite development and growth, as well as the host’s state of health [17].

Here, colonization behaviour in single and mixed infections of different regions of the *R. prolixus* intestinal tract are reported for the first time in order to better elucidate relevant variables that determine the distribution of the primary *T. cruzi* genotypes, TcI and TcII. The isolates used in the present study were obtained from distinct areas of Brazil with different landscapes and climatic conditions.

## Methods

### Parasites

The *T. cruzi* isolate MDID/BR/1993/C45 (C45/TcI) was obtained from the naturally infected marsupial host *Philander frenatus* from Teresópolis, Rio de Janeiro, south-east Brazil [18]. The second isolate, TBRA/BR/1999/JCA3 (JCA3/TcII), was recovered from naturally infected *Triatoma brasiliensis* from the João Costa municipality of Piauí, north-east Brazil. *T. cruzi* epimastigotes were grown in McNeal, Novy and Nicolle medium with a liver infusion tryptose overlay supplemented with 10% foetal calf serum [19]. After a few passages in culture medium in 1993 and 1999, respectively, both isolates were stored in liquid nitrogen for future use. The isolates were previously characterized as TcI and TcII by isoenzymatic analysis, lectin agglutination assays and polymerase chain reaction (PCR) analysis of the mini-exon gene, as well as the 24Sα and 18S rRNA regions [18,20-22].

### Insect feeding

A total of 120 *R. prolixus* fourth instar nymphs (L4) were reared at 26 ± 1°C at 60–70% relative humidity. For *T. cruzi* infections, three groups of insects were artificially fed through latex membranes using parasites (C45/TcI, JCA3/TcII and 50% C45/TcI + 50% JCA3/TcII) maintained in LIT culture medium near the end of their log phase, washed with phosphate buffered saline (pH 7.2) and then diluted in citrated, heat-inactivated rabbit blood [23-25]. The insects received a blood meal at a final concentration of 2 × 10^6^ parasites/mL. About 20 days later, the insects moulted to fifth instar nymphs (L5) and were fed 14 days afterward with parasite-free rabbit blood. Because some insects died or were not fully engorged, a final total of 90 L5 nymphs were used for the present analysis.

### Insect dissections

Dissections of 10 insects from each feeding group were performed at 7, 14 and 21 days after feeding (daf). After volume measurement, the parasite population density in the small intestine, rectal lumen and rectal wall, as well as densities of the metacyclic trypomastigote forms, were determined using Neubauer haemocytometers in a multiple field observation [24,25]. Numbers, percentages, means and standard deviations of the flagellates per insect were calculated [24]. Statistical analyses were performed using the Student’s *t*-test and one-way analysis of variance to evaluate the colonization patterns of the three *T. cruzi* groups infecting different *R. prolixus* intestinal regions and at different daf [24,26]. After analysis, the samples were used for genomic DNA extraction.

### Genomic DNA extraction

A 10-μL aliquot of each intestinal region (small intestine, rectal lumen and rectal wall) from each individual was collected at every daf time-point and pooled for DNA analysis. *T. cruzi* genomic DNA was extracted from the resulting 100-μL pooled samples using the Wizard SV Genomic DNA Purification System Kit (Promega, Madison, WI, USA) following the manufacturer’s protocol. The obtained DNA samples were precipitated, dried, resuspended in Milli-Q water to one-tenth of the original volume and then stored at −20°C for future use.

### Mini-exon PCR of different *R. prolixus* intestinal regions

Multiplex PCR was performed in triplicate using the primers described by Souto *et al*. [27]. The PCR-amplified products were resolved by electrophoresis on 2% agarose gels containing ethidium bromide and documented with a Kodak EDAS 290 gel documentation system (Kodak, Rochester, NY). The band intensities of the mixed infection samples were quantified using ImageJ ver. 1.48a imaging software (rsbweb.nih.gov/ij/). Only TcI and TcII values from the same lane were compared with each other. The means and standard deviations were calculated and statistically analysed as described above. For the analysis only not saturated PCR products were used.

## Results

### Parasite population density in the small intestine and rectal regions

**Figure 1:**
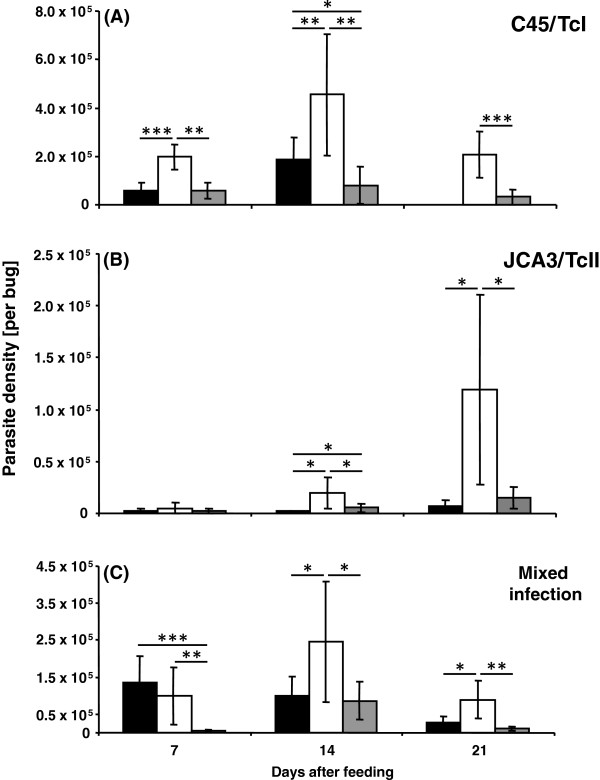
**Mean number of parasites of two different *T. cruzi *isolates in three intestinal regions (small intestine – black column; rectal lumen – white column and rectal wall – grey column) of *R. prolixus *(per bug) at different times after blood meal in single C45/TcI (A), JCA3/TcII (B) and mixed infection (C).** Standard deviation is shown for each analysed group. Statistically significant differences (* *p* < 0.05, ** *p* < 0.01, *** *p* < 0.001) are shown above the columns. (n = 10).

The *T. cruzi* infection rate of all three insect groups was 100%. The **C45/TcI** isolate colonized all three of the analysed intestinal regions (small intestine, rectal lumen and rectal wall), except at 21 daf when no parasites were detected in the small intestine (Figure 1A). At 7 daf the number of parasites in the rectal lumen (1.98 × 10^5^ cells/insect) was significantly higher than that in either the small intestine (0.58 × 10^5^ cells/insect, *p* < 0.001) or rectal wall (0.57 × 10^5^ cells/insect, *p* < 0.01), while no difference in density was found between the small intestine and rectal wall (Figure 1A). Also at 14 and 21 daf the parasites predominantly occupied the rectal lumen (4.55 × 10^5^ and 2.06 × 10^5^ cells/insect, respectively), particularly as compared to the rectal wall, which was colonized with a significantly lower number of flagellates (0.81 × 10^5^ and 0.33 × 10^5^ cells/insect, *p* < 0.01 and *p* < 0.001, respectively).

The **JCA3/TcII** isolate was mostly observed in the rectal lumen, mainly at 21 daf, achieving a concentration of 1.19 × 10^5^ cells/insect (Figure 1B). This isolate occupied all three intestinal regions, although in lower densities when compared to the TcI (Figure 1A) and mixed infection groups (Figure 1C). At daf 14, the rectal lumen (0.19 × 10^5^ cells/insect) and rectal wall (0.06 × 10^5^ cells/insect) contained significantly higher numbers of parasites (*p* < 0.05) than the small intestine (0.02 × 10^5^ cells/insect), whereas, at 21 daf, the number of parasites in the rectal lumen was significantly higher than that in the small intestine (0.07 × 10^5^ cells/insect, *p* < 0.05) and rectal wall (0.15 × 10^5^ cells/insect, *p* < 0.05).

In the **mixed infection** group the flagellates inhabited all of the studied intestinal regions, although in highly differing concentrations (Figure 1C). Some host intestinal regions contained a significantly higher number of parasites compared to the same regions in insects with single parasitic infections (Additional file 1: Table S1). At 7 daf, the numbers of parasites in the small intestine (1.4 × 10^5^ cells/insect) and rectal lumen (0.99 × 10^5^ cells/insect) were significantly higher (*p* < 0.001 and *p* < 0.01, respectively) than in the rectal wall region (0.05 × 10^5^ cells/insect). Also, at this time, the number of parasites in the small intestine was significantly higher than in both C45/TcI and JCA3/TcII (0.03 × 10^5^ cells/insect) single infection groups (*p* < 0.01 and *p* < 0.0001, respectively), whereas in the rectal lumen, the number of parasites in the mixed infection group was significantly lower (*p* < 0.01) than in the single C45/TcI infection group. At 14 daf, the parasite distribution pattern of the mixed infection was similar to the TcI-infected insect group, although there were significantly more flagellates (*p* < 0.05) in the small intestines of the C45/TcI group than in those of the mixed infection (0.99 × 10^5^ cells/insect) (Figure 1A, C). At this time, there were significantly fewer JCA3/TcII parasites in the small intestine (*p* < 0.0001), rectal lumen (*p* < 0.001) and rectal wall (*p* < 0.001) in comparison to the mixed infection group. At 21 daf, the number of parasites in the small intestine (0.27 × 10^5^ cells/insect) and rectal wall (0.11 × 10^5^ cells/insect) of the mixed infection group was significantly less than that in the rectal lumen (0.9 × 10^5^ cells/insect, *p* < 0.05 and *p* < 0.001, respectively).

### Population density of metacyclic trypomastigote forms

**Figure 2:**
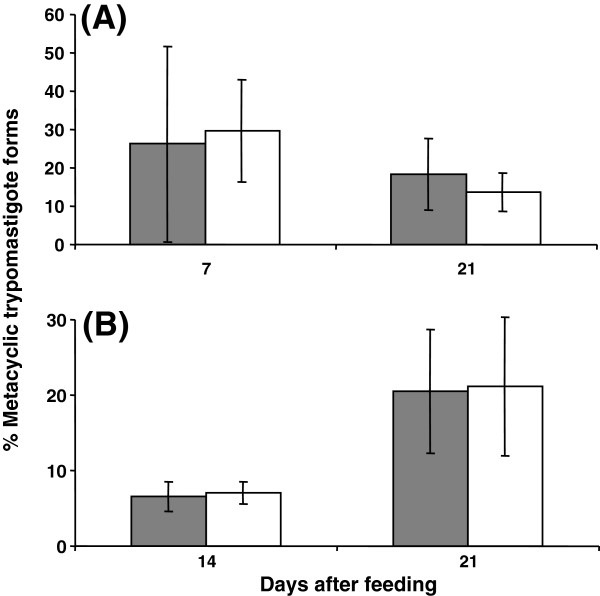
**Percentages of metacyclic trypomastigote *T. cruzi *forms in different intestinal regions (rectal lumen – grey column and rectal wall – white column) of *R. prolixus *at different times after the blood meal in single C45/TcI (A) and mixed infection (B).** Standard deviation is shown for each analysed group. (n = 10).

In the **C45/TcI** group similar percentages of metacyclic trypomastigote forms were detected at 7 daf in the rectal lumen and wall (26.2% and 29.6%, respectively; Figure 2A). At 21 daf, the percentage of metacyclic trypomastigote forms remained stable in the rectal lumen (18.3%), but significantly decreased in the rectal wall (13.7%, *p* < 0.05, Figure 2A).

In the **mixed infection** group at 14 daf the percentage of metacyclic trypomastigote forms in the rectal lumen and wall were almost identical (6.6% and 7.1%, respectively; Figure 2B). At 21 daf, the abundance of metacyclic trypomastigote forms significantly increased in both regions (20.5% and 21.1%, respectively; *p* < 0.01; Figure 2B). The C45/TcI and mixed infection groups presented similar percentages of metacyclic trypomastigote forms in the rectal lumen at 21 daf, in contrast to the rectal wall regions, where the mixed infection group showed a significantly (*p* < 0.05) higher percentage of metacyclic trypomastigote forms compared to the C45/TcI group (Figure 2B).

In the JCA3/TcII group, no metacyclic trypomastigote forms in any intestinal region were detected via haemocytometric analysis. In the small intestine of the C45/TcI at 14 daf and mixed infection at 7 daf metacyclic trypomastigote forms were only sporadically found. Therefore, these results were not included due to insufficient data for statistical analysis.

### *T. cruzi* detection in different digestive tract regions by mini-exon PCR

**Figure 3:**
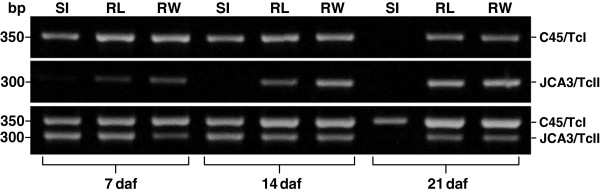
**Mini-exon PCR of samples obtained at 7, 14 and 21 daf from small intestine (SI), rectal lumen (RL) and rectal wall (RW) of *R. prolixus*.** (n = 10).

Mini-exon PCR with genomic DNA obtained from the pooled C45/TcI samples produced a 350-bp band, whereas PCR experiments using JCA3/TcII samples generated a 300-bp band. Thus, the two groups could be differentiated in different regions of the *R. prolixus* intestine. The obtained results demonstrated that the TcI group in a single infection could be detected in all studied intestinal tract regions of *R. prolixus*, except in the small intestine at 21 daf (Figure 3), corroborating the results of the parasite density in Figure 1A. In contrast to C45/TcI, JCA3/TcII mini-exon amplicon intensity increased at 21 daf (Figure 3), thereby also reflecting our microscopy results (Figure 1B). The lower abundance of parasites in some digestive tract regions of the TcII-infected insects was likely responsible for the weaker mini-exon bands or the absence of the respective PCR product. Two distinct bands were always observed using genomic DNA from the mixed infection group, except for that from the small intestine at 21 daf (Figure 3). Since both bands were amplified during the same PCR reaction, the intensities could be compared and quantified for each sample.

**Figure 4:**
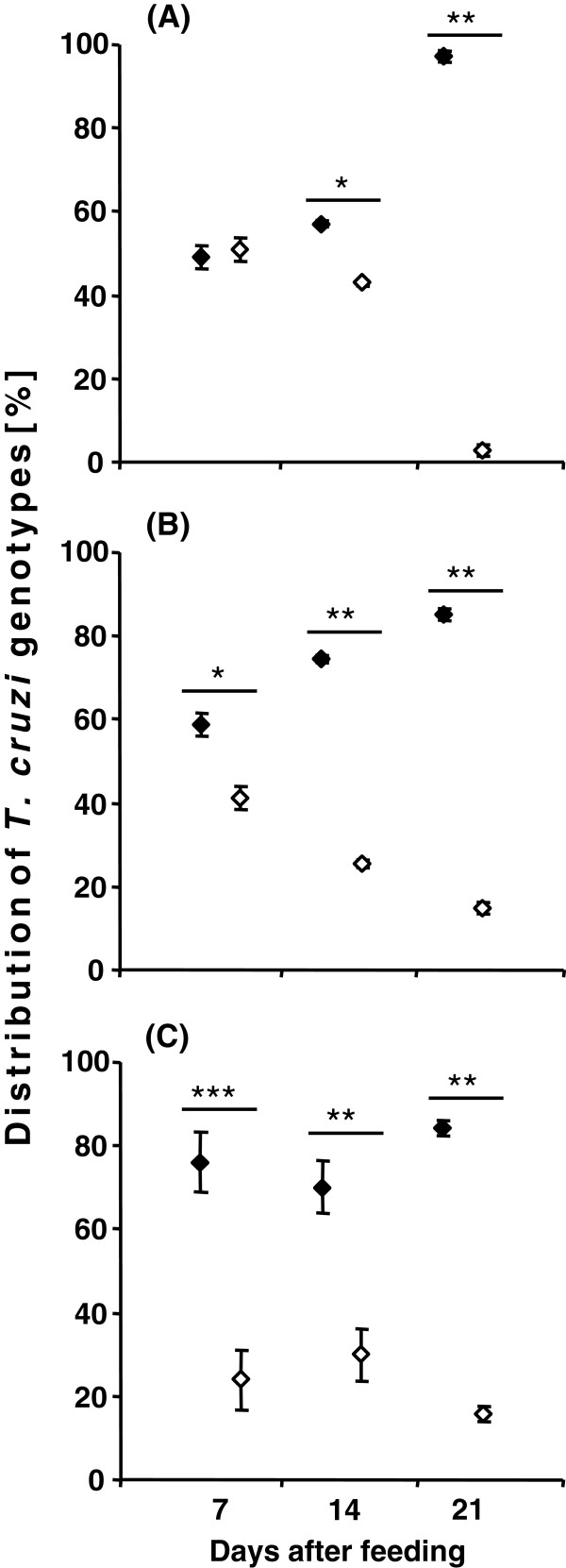
**Graphical illustration of the *T. cruzi *mini-exon amplicon intensity of mixed infections in different intestinal regions, small intestine (A), rectal lumen (B) and rectal wall (C) of *R. prolixus *mixed infections at daf, based on the results obtained by mini-exon PCR.** Standard deviation is shown for each analysed sample. Statistically significant differences (* *p* < 0.05, ** *p* < 0.01, *** *p* < 0.001) are shown above the values. (♦ - C45/TcI, ◊ - JCA3/TcII).

Quantification of the percentage of TcI and TcII, based on the mini-exon band intensity of the **mixed infection** and its statistical significance at the different daf in the intestinal regions are summarized in Figure 4. In the small intestine at 7 daf, the distribution of parasite populations was almost equal, but then significant differences were found at 14 and 21 daf (Figure 4A). The parasite distribution in the rectal lumen was similar to that of the small intestine but the C45/TcI population was already significantly predominant in comparison to JCA3/TcII at 7 daf (Figure 4B). In the rectal wall the distribution of the two parasite populations remained significantly different from 7 daf onward, in contrast to that seen in the small intestine and rectal lumen (Figure 4C).

## Discussion

Although the host immune response is vital to modulate the course of an infection, parasitic behaviour can be dramatically altered in the presence of other parasites or bacteria sharing the same host [24,28,29]. Remarkable differences in the number of parasites of each genotype were found among the experimental groups in the present study. In general, the total number of parasites was lower in the mixed infection group compared to that in the C45/TcI single infection group. One reason for the reduced number of parasites JCA3/TcII at 7 and 14 daf might have been caused by the slower growth of this isolate. However, while in the C45/TcI single infection group, parasites appeared only in the rectal lumen and wall at 21 daf, the differential PCR analysis found the presence of this isolate in the small intestine of mixed infection. By contrast, JCA3/TcII was present in the small intestine 21 daf when used as a single infection, but then vanished from this intestinal region in the mixed infection group. Thus, it seems that the dynamics of a parasitic infection is not only regulated by strain-specific growth behaviour but also by concomitant interactions. The *T. cruzi* genotype composition – mixed infections with genetically variable parasites – might also play an important role in parasitic development and transmission.

In a previous TcI and TcII mixed infection study with *T. brasiliensis*, a different parasitic distribution was reported [24]. Herein, two isolates obtained from distinct hosts and at different times post-infection were tested and revealed different colonization patterns between the mixed and single infection groups. At 20 daf, TcII (JCPD4) was highly abundant in the small intestine, but lower in the rectal lumen and completely absent in the rectal wall. Conversely, TcI (M1) was abundant in the rectal wall and lumen, but absent in the small intestine. In the mixed infection group, only the rectal wall and lumen were colonized, of which, the latter intestinal region had a higher degree of colonization. In this study both *T. cruzi* isolates produced metacyclic parasite forms. By contrast, in the present study JCA3/TcII was predominantly abundant in the rectal lumen and wall, although in different quantities, as well as higher concentrations in the rectal lumen, but yet lower in the rectal wall and did not produce metacyclic trypomastigote forms. The distribution of C45/TcI was similar to that verified in the *T. brasiliensis* study, which also applies to the quantity of the parasites. However, mini-exon PCR and the distribution analysis of both genotypes showed that the number of JCA3/TcII in the mixed infection was – except at 7 daf in the small intestine – significantly lower. Also striking was the absence of metacyclic trypomastigote forms in the JCA3/TcII infection group. Apparently, *T. brasiliensis* (Triatomini) is able to maintain and transmit both the TcI and TcII genotypes [24,25], whereas in *R. prolixus* (Rhodniini) only the TcI genotype was able to complete the life cycle. Even the unfamiliar MDID/BR/1994/C48 isolate (TcI), coming from another region and without prior contact to *T. brasiliensis* has been successfully maintained in this triatomine species and has shown high numbers of metacyclic trypomastigote forms in different intestinal regions [25]. By contrast, in the present study, even though *R. prolixus* was able to maintain the TcII isolate TBRA/BR/1999/JCA3 in its small intestine and rectum – even after moulting and 21 days after a second blood meal – no metacyclic trypomastigote forms were detected. A previous study showed that in the midgut of *R. prolixus*, the Y strain (TcII) was eliminated only a few days after infection [23]. Therefore, the TcII isolate used in the present study seems to be better adapted to *R. prolixus*, although currently available data are insufficient to assert that TcII is generally incompatible with *R. prolixus*. However, the fact that TcI was exclusively found in *R. prolixus* might be because of the fact that TcII is so far not adapted to this unfamiliar triatomine species [30,31]. Thus, the TcI genotype seems to be more generalistic, infecting various triatomine species, while TcII seems to be more specialized and highly adapted to certain triatomine species, such as *T. brasiliensis*. Importantly, these different biological relationships might be exploited to control Chagas disease.

The obtained molecular biology results widely reflected those of our microscopic analysis, except for the JCA3/TcII colonization of the small intestine at 21 daf from which no mini-exon fragment could be amplified. This contradiction might be related to the low colonization and non-homogenous parasitic distribution in the small intestine and, thus, their loss during the insect dissection procedure and sample preparation. Another influencing factor might have been the lower sensitivity of the mini-exon PCR in comparison to other methods used for *T. cruzi* infection diagnostics [32]. Thus, the mini-exon PCR was effective in the present study only after concentrating the extracted genomic DNA 10-fold.

The distribution of *T. cruzi* subpopulations in nature can best be explained by the selection of the parasite by the vertebrate and invertebrate hosts, based on the parasitic growth rates and other selective pressures, such as the host immune response and physiological differences between the organisms [33]. Bosseno *et al*. [34] showed that *T. cruzi* isolates of different genotypes drastically lost diversity after passage in axenic medium, whereas triatomines were clearly more competent to maintain mixed *T. cruzi* infections [35,36]. In the present paper *R. prolixus* was not only able to maintain C45/TcI but also the unfamiliar JCA3/TcII isolate, which originated from another triatomine species (*T. brasiliensis*) from a distant region. However, to maintain a parasitic infection in the midgut is only one part of its natural development, as for completion of its life cycle, metacyclogenesis is crucial. Indeed, in JCA/TcII infected *R. prolixus* no metacyclic trypomastigote forms were found, only epimastigotes, and *T. cruzi* epimastigotes are not able to infect a vertebrate host. For production of metacyclic trypomastigote forms, the epimastigote stage attaches to the perimicrovillar membranes of the posterior midgut or rectal cuticle and a population change occurs, thereafter metacyclic trypomastigote forms are washed out with the faeces and thus are able to infect a new vertebrate host [37]. Usually, the attachment to the small intestine and rectum epithelium is associated with the metacyclogenesis rate [37,38]. Our results also indicate a role for the substrate in the attachment rate of *T. cruzi*. Specifically, the number of parasites was balanced and segregated in the course of infection at 7 daf in both lumen of the small intestine and rectum. In the rectal wall the C45/TcI isolate was dominant over that of the JCA3/TcII isolate from the beginning of the analysis. Therefore, we have to ask whether different *T. cruzi* genotypes are able to differentiate between Rhodniini and Triatomini intestinal epithelia. The abundance and absence of metacyclic trypomastigote *T. cruzi* forms of C45/TcI and JCA3/TcII, respectively, might be based on the lower efficiency of the latter to adhere to the intestine of *R. prolixus*. Indeed, previous studies have demonstrated the influence of surface components from the triatomine midgut and rectum on *T. cruzi* development [38,39]. Intrinsic factors, such as cruzipain or glycoinositolphospholipids, seem to be crucial for the development of metacyclic trypomastigote forms [40,41]. For instance, the addition of exogenous cruzipain reportedly increased the metacyclogenesis rate of transgenic chagasin-overexpressing *T. cruzi*[41]. To develop full activity such factors require specific conditions in the intestinal environment of the host insects, which likely differ from species to species. At 21 daf the percentage of metacyclic trypomastigote forms in the rectal wall of the host insects was significantly higher in the mixed infection group compared to the single C45/TcI infection group. Therefore, it is possible that in mixed infections, two different *T. cruzi* genotypes cooperate with each other so that metacyclogenesis is enhanced.

## Conclusions

The present study characterized the colonization profiles of two *T. cruzi* isolates (TcI, MDID/BR/1993/C45 and TcII, TBRA/BR/1999/JCA3) in different regions of the *R. prolixus* intestinal tract. This analysis showed that the C45/TcI isolate was better adapted to *R. prolixus* than JCA3/TcII, as indicated by the greater parasitic colonization. Only in the single C45/TcI infection and mixed infection groups, respectively, metacyclic trypomastigote forms were found in the host rectums. JCA3/TcII was able to survive in *R. prolixus* over a long period – even when competing with C45/TcI in the mixed infection scenario, but did not produce metacyclic trypomastigote forms. Since this genotype was shown to be able to complete its life cycle in other triatomine species, we must conclude that the different environments or inadequate production of intrinsic factors were responsible for the growth differences observed in the JCA3/TcII infection model. Furthermore, these results indicate that the distribution of *T. cruzi* throughout South America is likely dependent on the abundance of suitable vectors.

## Competing interests

The authors declare that they have no competing interests.

## Authors’ contributions

CACA designed the study protocol, developed and carried out the experiments, discussed and drafted the manuscript; PJW was responsible for the statistical analysis and figures, participated in the development of the experiments and in the discussion of the data; AMJ developed and suggested this study, corrected and discussed the manuscript. All authors read and approved the final version of the manuscript and are guarantors of the paper.

## Supplementary Material

Additional file 1: Table S1 Statistical differences on parasite densities between the C45/TcI, JCA3/TcII single and the mixed infection group. (n.s. - not significant, n.d. - not defined because at 21 daf in the C45/TcI infection no parasites were found in the small intestine, because of this lack of data statistical analysis was not carried out). Click here for file
