# Supplementary material for: TcI/TcII co-infection can enhance Trypanosoma cruzi growth in Rhodnius prolixus
Source: Parasit Vectors. 2014 Mar 4;7:94. doi: 10.1186/1756-3305-7-94 (PMC4015778; doi:10.1186/1756-3305-7-94)
Supplement: Additional file 1: Table S1 — Statistical differences on parasite densities between the C45/TcI, JCA3/TcII single and the mixed infection group. (n.s. - not significant, n.d. - not defined because at 21 daf in the C45/TcI infection no parasites were found in the small intestine, because of this lack of data statistical analysis was not carried out). [file 1756-3305-7-94-S1.doc]

Additional table: Statistical differences on parasite densities between the C45/TcI, JCA3/TcII single and the mixed infection group. (n.s. - not significant, n.d. - not defined because at 21 daf in the C45/TcI infection no parasites were found in the small intestine, because of this lack of data statistical analysis was not carried out)

| **Compared groups** | **7 daf** | | | **14 daf** | | | **21 daf** | | |
| --- | --- | --- | --- | --- | --- | --- | --- | --- | --- |
|  | **SI** | **RL** | **RW** | **SI** | **RL** | **RW** | **SI** | **RL** | **RW** |
| **C45/TcI-Mix** | ***p*<0.01^1^** | ***p*<0.01^2^** | **n.s.** | ***p*<0.05^2^** | **n.s.** | **n.s.** | **n.d.** | ***p*<0.05^2^** | **n.s.** |
| **JCA3/TcII-Mix** | ***p*<0.0001^1^** | ***p*<0.01^1^** | **n.s.** | ***p*<0.0001^1^** | ***p*<0.001^1^** | ***p*<0.001^1^** | **n.d.** | **n.s.** | **n.s.** |

^1^significant higher number of parasites in the mixed infection

^2^significant lower number of parasites in the mixed infection
